# Enhancement of mucosal innate and adaptive immunity following intranasal immunization of mice with a bovine adenoviral vector

**DOI:** 10.3389/fimmu.2023.1305937

**Published:** 2023-11-21

**Authors:** Ekramy E. Sayedahmed, Nelly O. Elshafie, GuangJun Zhang, Sulma I. Mohammed, Suryaprakash Sambhara, Suresh K. Mittal

**Affiliations:** ^1^ Department of Comparative Pathobiology, Purdue Institute for Immunology, Inflammation and Infectious Diseases, and Purdue University Center for Cancer Research, College of Veterinary Medicine, Purdue University, West Lafayette, IN, United States; ^2^ Influenza Division, Centers for Disease Control and Prevention, Atlanta, GA, United States

**Keywords:** innate immunity, mucosal immunity, mucosal immunization, bovine adenoviral vector, human adenoviral vector, TLR, cytokine, chemokine

## Abstract

**Introduction:**

Nonhuman adenoviral (AdV) gene delivery platforms have significant value due to their ability to elude preexisting AdV vector immunity in most individuals. Previously, we have demonstrated that intranasal (IN) immunization of mice with BAd-H5HA, a bovine AdV type 3 (BAdV3) vector expressing H5N1 influenza virus hemagglutinin (HA), resulted in enhanced humoral and cell-mediated immune responses. The BAd-H5HA IN immunization resulted in complete protection following the challenge with an antigenically distinct H5N1 virus compared to the mouse group similarly immunized with HAd-H5HA, a human AdV type 5 (HAdV5) vector expressing HA.

**Methods:**

Here, we attempted to determine the activation of innate immune responses in the lungs of mice inoculated intranasally with BAd-H5HA compared to the HAd-H5HA-inoculated group.

**Results:**

RNA-Seq analyses of the lung tissues revealed differential expression (DE) of genes involved in innate and adaptive immunity in animals immunized with BAd-H5HA. The top ten enhanced genes were verified by RT-PCR. Consistently, there were transient increases in the levels of cytokines (IL-1α, IL-1β, IL-5, TNF- α, LIF, IL-17, G-CSF, MIP-1β, MCP-1, MIP-2, and GM-CSF) and toll-like receptors in the lungs of the group inoculated with BAdV vectors compared to that of the HAdV vector group.

**Conclusion:**

These results demonstrate that the BAdV vectors induce enhanced innate and adaptive immunity-related factors compared to HAdV vectors in mice. Thus, the BAdV vector platform could be an excellent gene delivery system for recombinant vaccines and cancer immunotherapy.

## Introduction

Adenoviruses (AdV) are icosahedral non-enveloped viruses with 25-48 kb dsDNA genomes (1). AdV vector-based vaccines are capable of eliciting both humoral and cell-mediated immune (CMI) responses (2, 3) by stimulating innate immunity through both Toll-like receptor (TLR)-mediated and TLR-independent pathways (4, 5). Unlike subunit or inactivated virus vaccines, Adv vector-based vaccines do not require an adjuvant for their immunogenicity. AdV vector-based influenza vaccines have shown immense promise in eliciting protective immunity in animal models (6, 7) and human clinical trials (8, 9). Also, Adv vectors are excellent delivery vehicles for cancer gene therapy (10–12).

Due to the potential presence of more than 100 AdV types in humans, there is a high possibility of developing Adv-specific neutralizing antibodies, known as ‘preexisting vector immunity, in the general population (13–15). This vector immunity could adversely impact the efficacy of several human Ad (HAdV) vector-based delivery systems. Hence, several nonhuman Ads have been developed as gene delivery vectors to avoid vector immunity (16, 17). These nonhuman Ad vectors can be based on bovine AdV (BAdV), simian AdV, ovine AdV, canine AdV, porcine AdV, avian AdV, or murine AdV (16–18).

We have shown that the BAdV3 vector system can induce humoral and CMI responses against HA of an H5N1 influenza virus even in exceptionally high levels of HAdV vector immunity (19). Moreover, preexisting HAdV-neutralizing antibodies in humans do not cross-neutralize BAdV3 (20), and HAdV-specific CMI response does not cross-react with BAdV3 (5). BAdV3 internalization into the cells is independent of the HAdV5 receptors [Coxackievirus-adenovirus receptor (CAR) and αvβ3 or αvβ5 integrin] (21); however it utilizes α(2,3)-linked and α(2,6)-linked sialic acid-binding proteins as major receptors for internalization (22). BAdV3 efficiently transduces the heart, kidney, lung, liver, and spleen. The vector persists longer than a HAdV5 vector, especially in the heart, kidney, and lung in a mouse model (23). Sequential administration of HAdV5 and BAdV3 vectors overcomes vector immunity in an immunocompetent mouse model of breast cancer (20), and the persistence of the BAdV3 genome in human and nonhuman cell lines is similar to HAdV5 vectors (24). Therefore, BAdV3 vectors offer an attractive replacement to HAdV vectors for circumventing high levels of preexisting HAdV immunity with comparable safety profiles as HAdV vectors.

In an earlier study, we demonstrated that the BAd-H5HA, a BAdV vector expressing hemagglutinin (HA) of the H5N1 influenza virus, elicited significantly better immune responses compared to HAd-H5HA, even at a reduced dose (25). To understand the factors responsible for the antigen-specific enhanced immune responses with the BAdV vector-based platform, we employed transcriptome analyses of the lung tissues from BAd-H5HA-inoculated mouse groups and compared with HAd-H5HA-inoculated groups. The genes involved in innate and adaptive immune responses were highly expressed in BAd-H5HA-inoculated groups. The top ten of these enhanced genes involved in innate and adaptive immunity were validated by qRT-PCR analyses. In addition, the upregulation of Toll like receptor genes (TLR2, TLR3, TLR4, TLR7, and TLR9) in the lungs compared to the HAdV vector was also confirmed by qRT-PCR. Furthermore, some of these highly expressed genes related to innate and/or adaptive immune responses-related molecules or cytokines were verified by multiplex assay. Our results suggest that higher expression of factors associated with innate and adaptive immune responses could be critical in eliciting better immune responses in animals immunized with the BAdV vector platform compared to the HAdV vector-based delivery system.

## Results

### Differentially expressed genes in the lungs of BAd-H5HA-inoculated mice compared to that of the HAd-H5HA-inoculated group

Previously, we have shown that IN (intranasal) immunization of mice with BAd-H5HA induced enhanced humoral and CMI responses compared to the group vaccinated similarly with HAd-H5HA, even with a reduced vaccine dose (25). We hypothesized that enhanced expression of immune response-related genes might be a critical difference between BAd-H5HA and HAd-H5HA. To test this hypothesis, we collected lung tissues at 6, 12, 24, and 48 h post-infection (PI) from mouse groups infected with BAd-H5HA, HAd-H5HA, or PBS (**Table 1**). Total RNA extracted from the lung tissues was used for RNA-Seq analyses to uncover DE genes.

**Table 1:** Study design.

	Mouse 1	Mouse 2	Description
1	PBS1-1	PBS2-1	PBS intranasal for 6 h
2	PBS1-2	PBS2-2	PBS intranasal for 12 h
3	PBS1-3	PBS2-3	PBS intranasal for 24 h
4	PBS1-4	PBS2-4	PBS intranasal for 48 h
5	HHA1-1	HHA2-1	HAd-HA vector intranasal for 6 h
6	HHA1-2	HHA2-2	HAd-HA vector intranasal for 12 h
7	HHA1-3	HHA2-3	HAd-HA vector intranasal for 24 h
8	HHA1-4	HHA2-4	HAd-HA vector intranasal for 48 h
9	BHA1-1	BHA2-1	BAd-HA vector intranasal for 6 h
10	BHA1-2	BHA2-2	BAd-HA vector intranasal for 12 h
11	BHA1-3	BHA2-3	BAd-HA vector intranasal for 24 h
12	BHA1-4	BHA2-4	BAd-HA vector intranasal for 48 h

BALB/c mice (2 animals/group) were inoculated intranasally with PBS (Mock) or 3×10^7^ PFU of HAd-H5HA or BAd-H5HA. At 6, 12, 24, and 48 h post-inoculation, two animals/group were euthanized under anesthesia, and lung samples were collected for RNA extraction.

**Figure 1:**
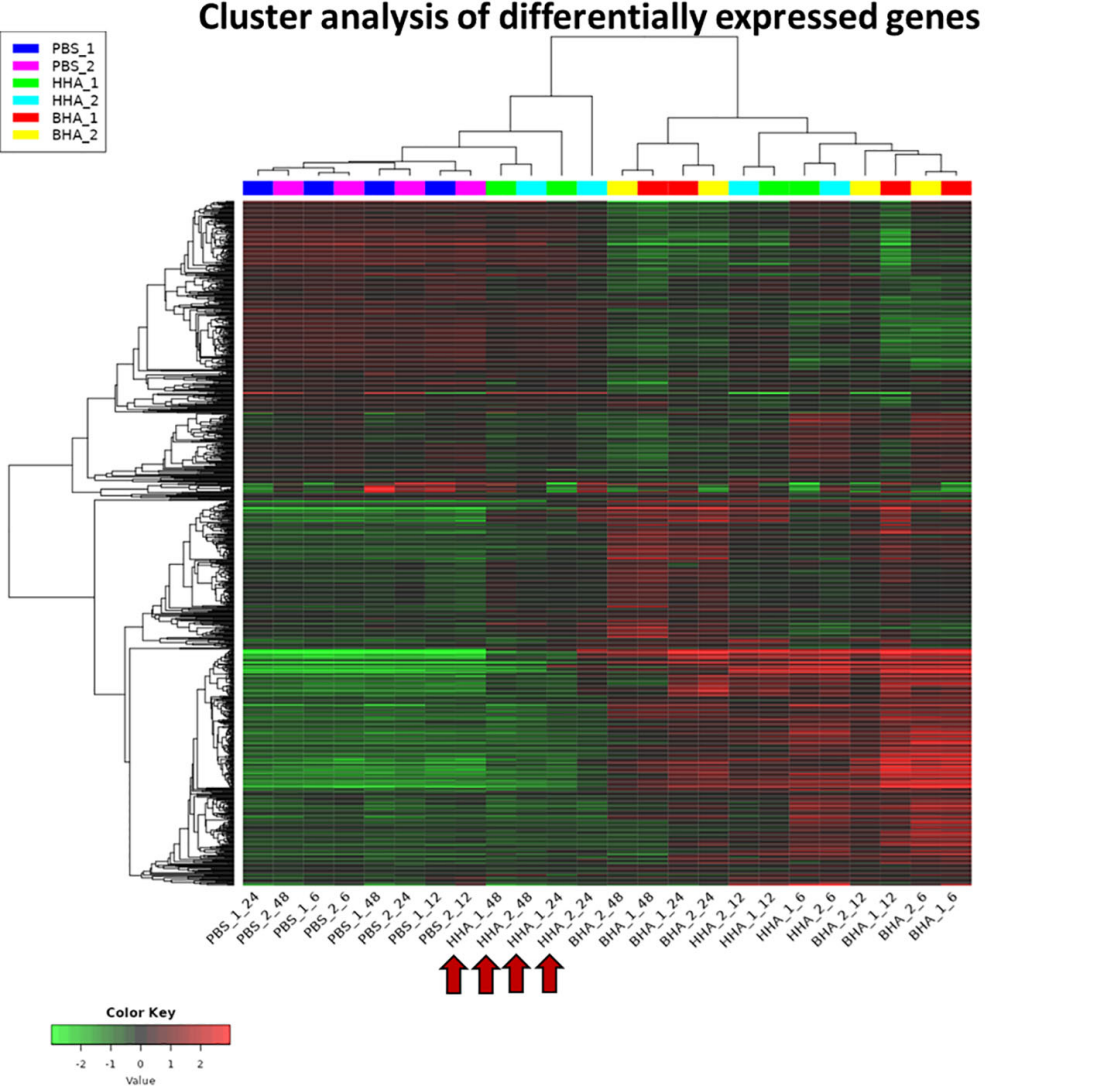
A heatmap uncovering the differential expressed (DE) genes in the lungs of mice at various times post-inoculation (PI) with BAd-H5HA (BHA), HAd-H5HA (HHA), or mock (PBS). Two independent samples for each group are shown, depicting an increase or decrease in expression levels, with red or green color, respectively. PBS_1_06, PBS_1_12, PBS_1_24, & PBS_1_48 represent 6, 12, 24, and 48 h PI samples from mouse #1 in mock-inoculated group. HHA_1_06, HHA_1_12, HHA_1_24, & HHA_1_48 represent 6, 12, 24, and 48 h PI samples from mouse #1 in HAd-H5HA-inoculated group. BHA_1_06, BHA_1_12, BHA_1_24, & BHA_1_48 represent 6, 12, 24, and 48 h PI samples from mouse #1 in BAd-H5HA-inoculated group. The same time points are repeated with mouse #2. The red arrows are showing the later stages (24 and 48 h) of HAd-H5HA groups gene expression clustering close to the PBS basal group.

A heat map of RNA-Seq data is shown where the values of data points are represented to visualize gene expression levels (**Figure 1**). Each row represents a gene, each column represents a sample, and a color gradient indicates the expression level of each gene. The heat map shows the clusters of genes that are co-regulated and the samples with similar expression profiles. The PBS-treated groups clustered together at 6, 12, 24, or 48 h (basal group), indicating the homogeneity of mock samples. While both BAd-H5HA and HAd-H5HA treated groups at 6 and 12 h form separated clusters from the mock samples (**Figure 1**). More interestingly, at the later stages (24 and 48 h), BAd-H5HA and HAd-H5HA groups showed a distinct gene expression profile, where the BAd-H5HA groups clustered mainly with the early states (6 h and 12 h), but the HAd-H5HA groups were clustered with the PBS basal group annotated with arrows (**Figure 1**). This difference suggests that the HAd-H5HA-induced DE gene expression levels decline rapidly. In contrast, the BAd-H5HA-induced DE maintained its overexpression status for a longer period.

**Figure 2:**
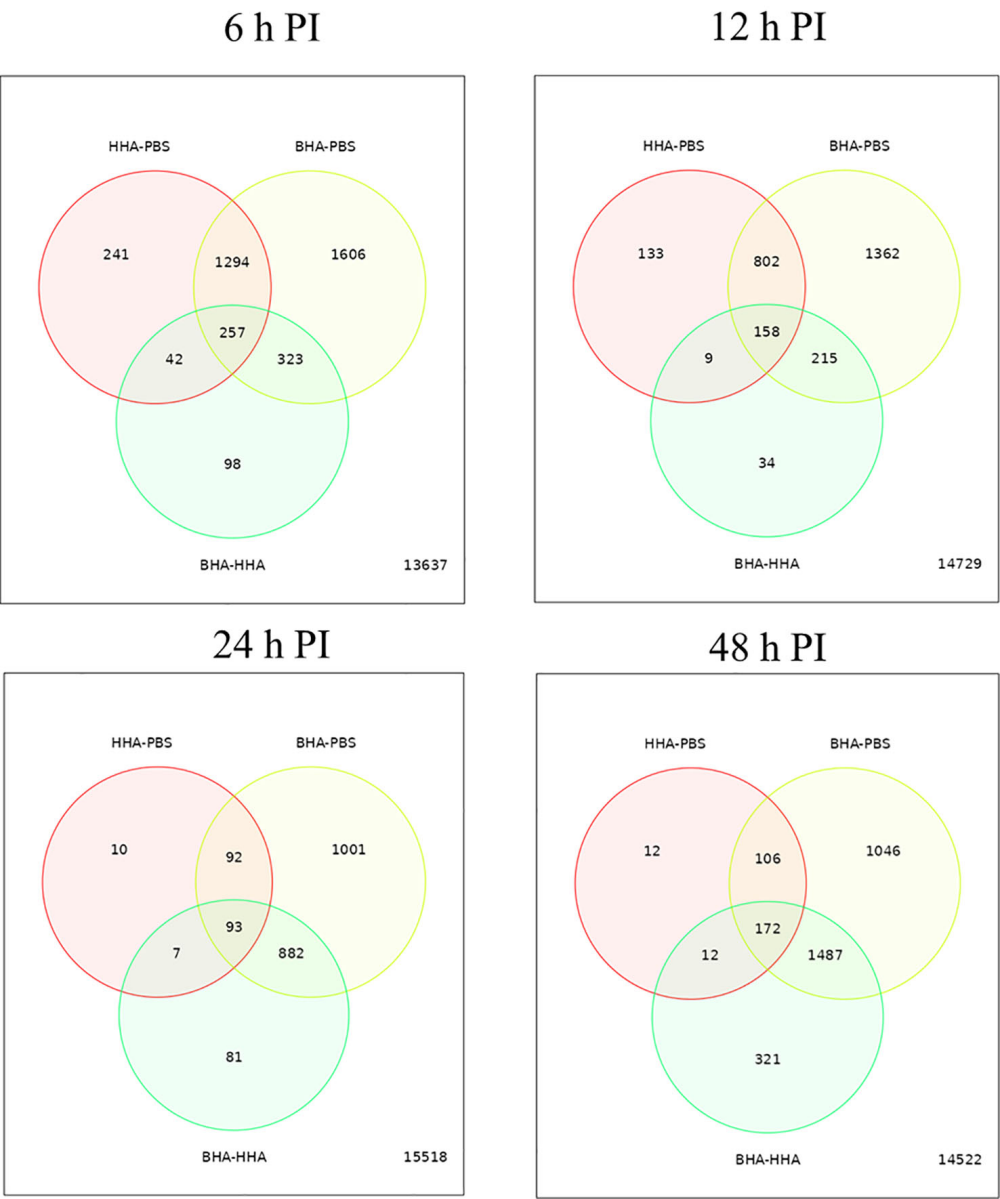
Venn diagram of differentially expressed (DE) genes in the lungs of mice at 6, 12, 24, and 48 h post-inoculation (PI) with BAd-H5HA (BHA), HAd-H5HA (HHA) or mock (PBS). The red circle represents the number of differentially expressed genes (DEGs) in the HAd-H5HA(HHA) compared to the PBS group. The yellow circle represents the number of DEGs in the BAd-H5HA (BHA) compared to the PBS group. The green circle represents the number of DEGs in the BAd-H5HA (BHA) compared to HAd-H5HA (HHA) group. The overlapping region between the circles indicates the number of DE genes shared between the groups.

To determine variability in the expression of DE genes in the BAd-H5HA or the HAd-H5HA group, a graphical representation by Venn diagram analysis was examined to infer the overall distribution of DE genes between the vaccine groups at 6, 12, 24, and 48 h PI (**Figure 2**). The average DE genes within the group was used to compare the gene expression patterns between samples. The overlapping region between the circles indicates the number of DE genes shared between the groups. Compared to the PBS group, the BAd-H5HA (BHA) group shows the highest number of DE genes at all times than the HAd-H5HA (HHA) group. The BAd-H5HA group shows variable numbers of DE genes compared to the HAd-H5HA group at all time points.

**Figure 3:**
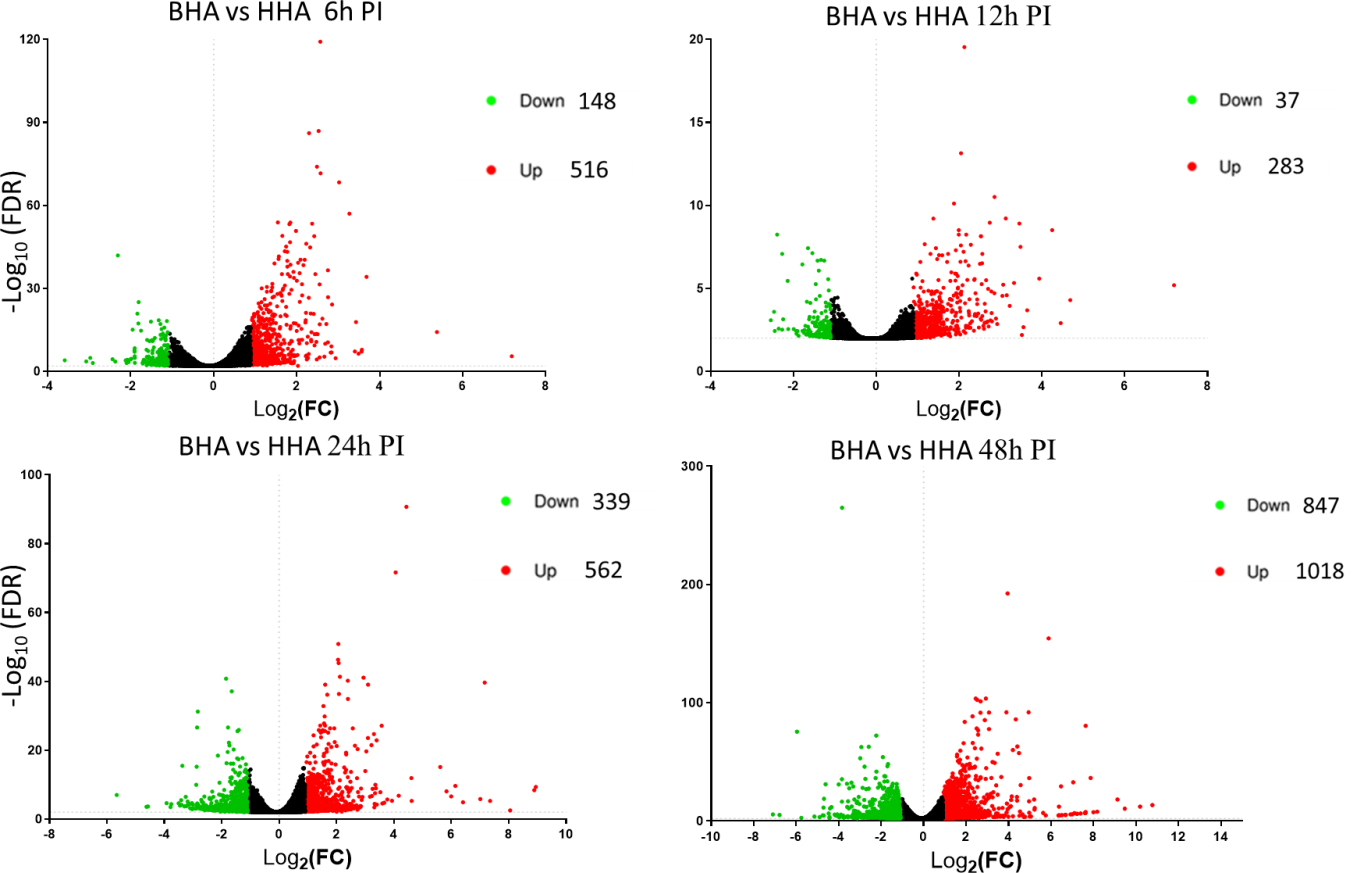
Volcano plots from the DESeq2 analysis of differentially expressed (DE) genes in the lungs of mice at 6, 12, 24, and 48 h post-inoculation (PI) with BAd-H5HA (BHA), HAd-H5HA (HHA), or mock (PBS). Log2 fold changes are plotted on the x-axis, and -log10 p-values on the y-axis. Each point represents a gene, the red color dots representing the upregulated genes, and the green color dots representing the downregulated genes. The cutoff of the log2 fold change is 2.5, and the cutoff of the -log10 (FDR<0.05) is 1.3.

Further in Volcano plot analysis, there were 664, 320, 901, and 1865 DE genes at 6, 12, 24, and 48 h PI, respectively, between BAd-H5HA and HAd-H5HA groups (**Figure 3**). The overall gene expression profiles are similar at 6 h and 12 h, while quite different at 24 h and 48 h time points. Our data suggest that host responses to BAd-H5HA and HAd-H5HA are somewhat similar at early stages (6 and 12 h PI) than the later stages (24 and 48 h PI). The top ten overexpressed genes in the BAd-H5HA group compared to the HAd-H5HA group at 6, 12, 24, and 48 h PI are listed (**Supplementary Table 2**).

**Figure 4:**
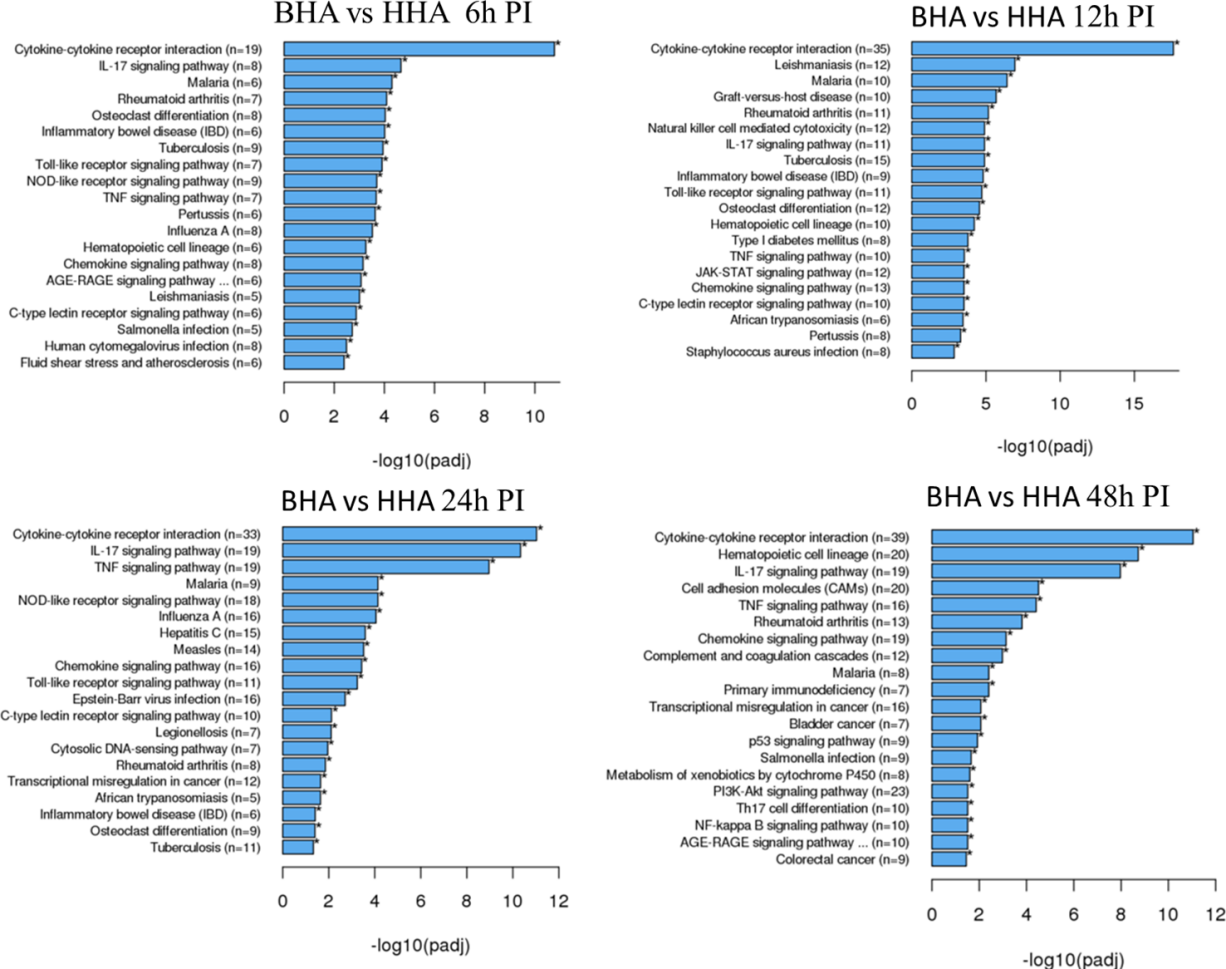
KEGG pathways highlighting differentially expressed (DE) groups of genes within the same biological pathway in the lungs of mice at 6, 12, 24, and 48 h post-inoculation (PI) with BAd-H5HA (BHA) compared to the HAd-H5HA (HHA) groups.

**Figure 5:**
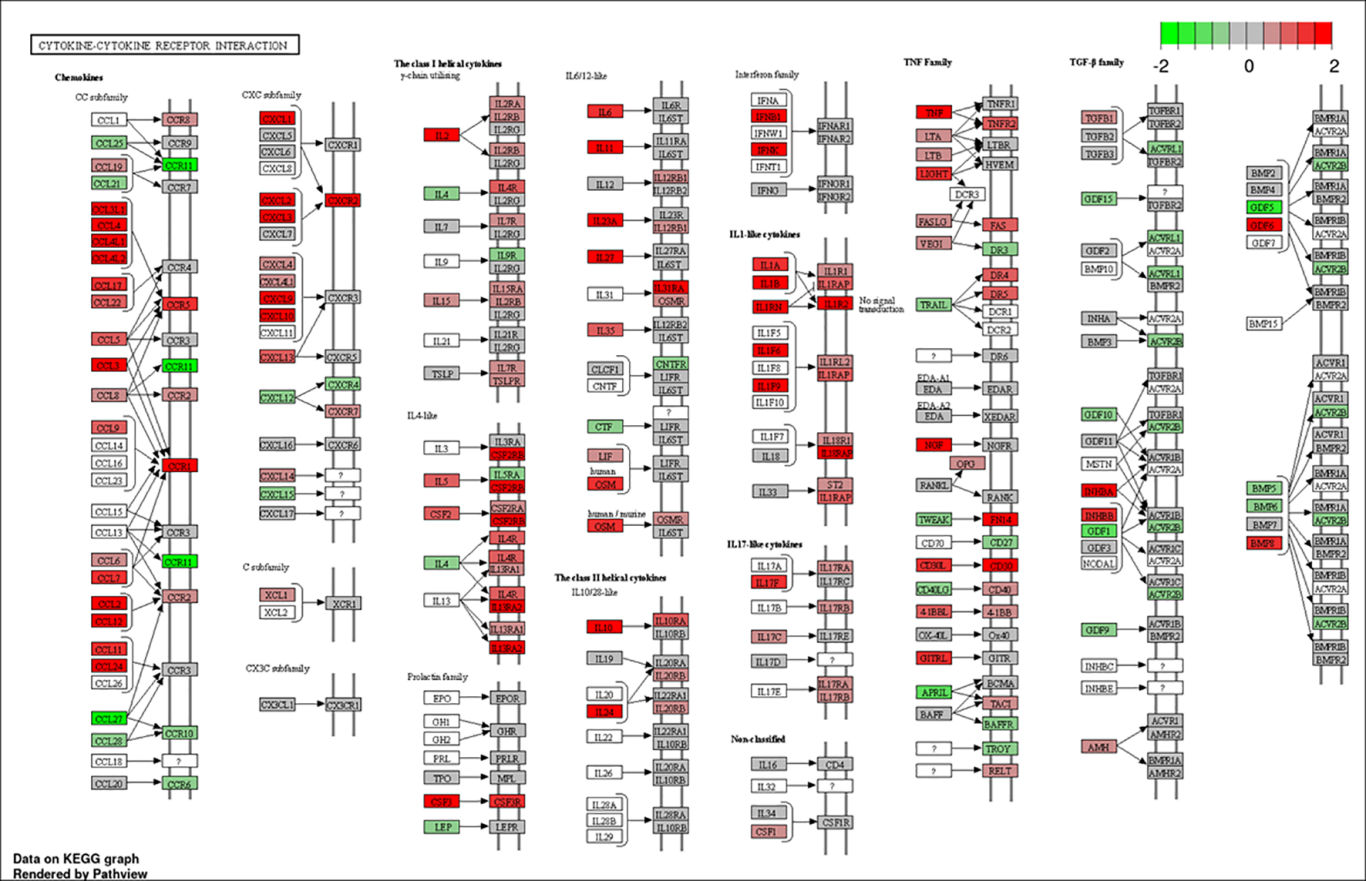
Cytokine-cytokine receptor interaction pathway. DE genes KEGG pathway analysis with significance cutoff (FDR) 0.1 was performed for BAd-H5HA versus HAd-H5HA. Red and green represent up-regulated and down-regulated genes, respectively. The KEGG database pathway analysis with a false discovery rate (FDR) 0.1 cutoff value for significance was used to show the top 10 pathways.

To better understand the biological functions of the DE genes, KEGG (Kyoto Encyclopedia of Genes and Genomes) pathway analyses were conducted for the BAd-H5HA group compared to the HAd-H5HA group at 6, 12, 24, and 48 h PI. The primary biological pathways relevant to the innate and adaptive immune responses that were highlighted include cytokine-cytokine receptor interaction (19 genes), IL-17 signaling pathways (8 genes), TLR signaling pathway (7 genes), NOD-like receptor signaling pathway (9 genes), and TNF signaling pathway (7 genes) at 6 h PI; cytokine-cytokine receptor interaction (35 genes), natural killer cell-mediated cytotoxicity (12 genes), IL-17 signaling pathways (11 genes), TLR signaling pathway (11 genes), and TNF signaling pathway (10 genes) at 12 h PI; cytokine-cytokine receptor interaction (33 genes), IL-17 signaling pathways (19 genes), TNF signaling pathway (19 genes), NOD-like receptor signaling pathway (18 genes), chemokine signaling pathway (16 genes), and TLR signaling pathway (11 genes) at 24 h PI; and cytokine-cytokine receptor interaction (39 genes), hematopoietic cell linkage (20 genes), IL-17 signaling pathways (19 genes), cell adhesion molecules (20 genes), TNF signaling pathway (16 genes), and chemokine signaling pathway (19 genes), at 48 h PI (**Figure 4**). Overall, our transcriptome study of DE genes suggests that the major signaling pathways that were upregulated included cytokine-cytokine receptor interaction, IL-17 signaling pathway, TLR signaling pathway, TNF signaling pathway, and NOD-like receptor signaling pathway. The top 10 pathways by KEGG database pathway analysis are listed (**Table 2**). The cytokine-cytokine receptor interaction pathway has the highest number of DE genes (228). This pathway has multiple chemokine and cytokine genes upregulated in the BAd-H5HA group than the HAd-H5HA group (**Figure 5**).

**Table 2:** KEGG top 10 pathways analysis of BAd-H5HA DE genes than HAd-H5HA.

Direction	GAGE analysis: BAd-H5HA vs. HAd-H5HA	statistic	Genes	adj. P-value
Down	Drug metabolism	-4.7087	44	1.60E-03
	Metabolism of xenobiotics by cytochrome P450	-4.1298	45	6.80E-03
Up	Cytokine-cytokine receptor interaction	6.0313	228	6.30E-07
	NOD-like receptor signaling pathway	4.814	150	1.90E-04
	Viral protein interaction with cytokine and cytokine receptor	4.6804	79	2.70E-04
	Coronavirus disease	4.6203	203	2.70E-04
	TNF signaling pathway	4.5248	108	3.30E-04
	IL-17 signaling pathway	4.4162	83	5.00E-04
	NF-kappa B signaling pathway	3.8989	99	3.10E-03
	Toll-like receptor signaling pathway	3.7856	83	4.50E-03

The KEGG database pathway analysis with a false discovery rate (FDR) 0.1 cutoff value for significance was used to show the top 10 pathways.

### Validation of top ten upregulated genes by BAdV vector

To further validate our identified DE genes by RNA-Seq, we examined the top ten upregulated genes (**Supplementary Table 2**) by examining RNA extracted from the lung tissues of BAd-H5HA- or HAd-H5HA-inoculated mice using qRT-PCR. Indeed, all the 10 genes were upregulated in BAd-H5HA groups compared to HAd-H5HA groups at 6, 12, 24, and 24 h PI (**Figure S1A**). The gene ontology (GO) biological processes of these DE genes showed highly enriched natural killer cell pathways (**Figure S1B**). The KEGG pathway analysis indicated the involvement of four genes in the cytokine-cytokine receptors interaction, two genes in the TLR process, and two genes in viral protein interaction with cytokine and cytokine receptor interaction beside other pathways (**Figure S1C**). A tree map (**Figure S1D** right) and network map (**Figure S1D** left) illustrated potential interactions of different KEGG pathways.

### Upregulation of TLR genes by BAdV vector

AdV plays a vital role in activating TLR-mediated innate immunity (4). To ascertain enhanced expression of TLR genes in the lungs of the BAd-H5HA-inoculated mouse group compared to the HAd-H5HA group, qRT-PCR analyses were performed. The qRT-PCR analyses showed that the BAdV groups at various time points have higher expression levels of TLR2, TLR3, TLR4, TLR7, and TLR9 than the HAdV groups (**Figure S2**).

### Expression of innate and adaptive immunity-related factors in the lungs

**Figure 6:**
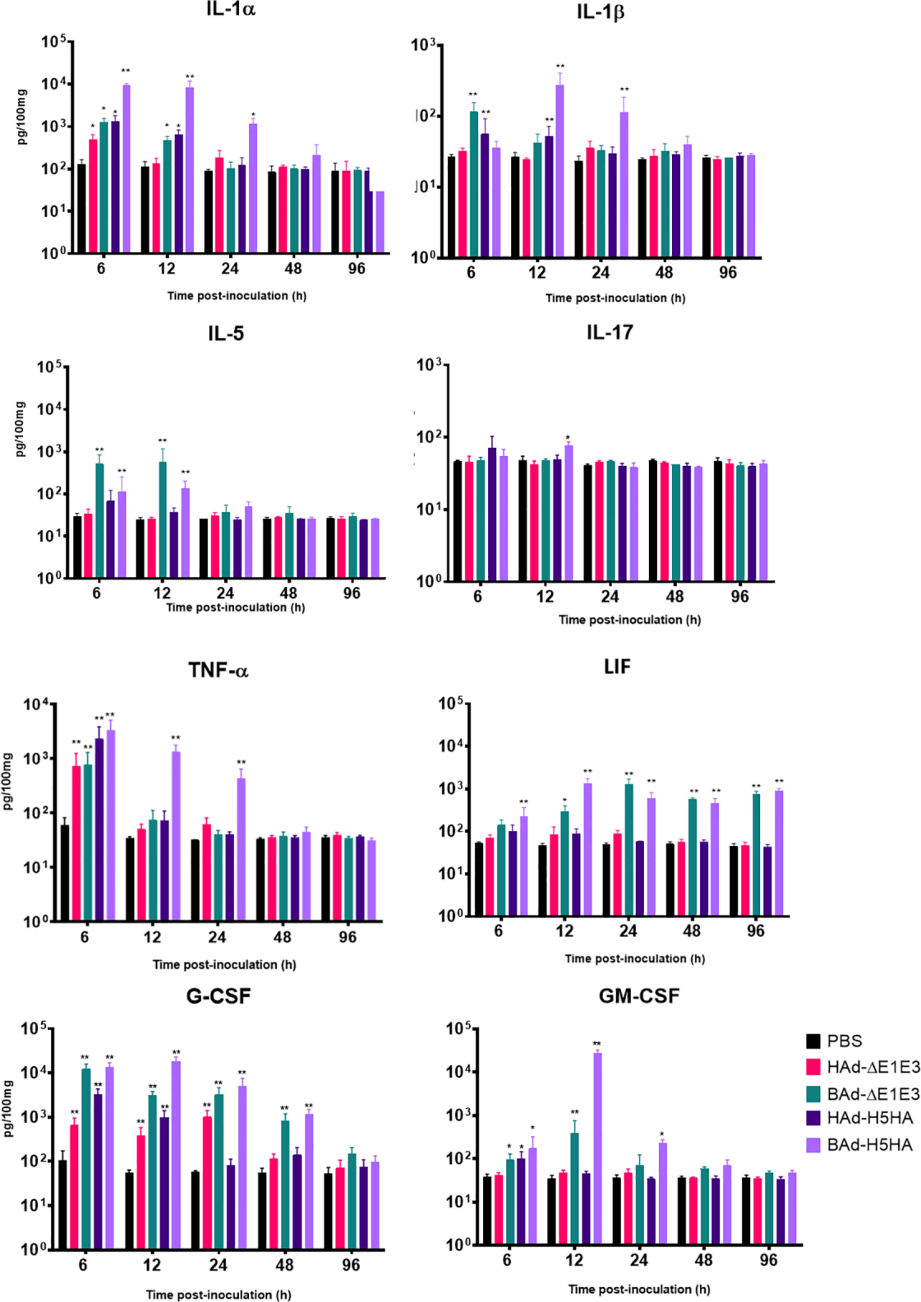
Innate cytokine multiplex assay showing the increase in cytokines levels in BAdV vectors inoculated groups. BALB/c mice (3 animals/group) were inoculated IN once with PBS or with 3 × 10^7^ PFU per animal of HAd-ΔE1E3, BAd-ΔE1E3, HAd-H5HA or BAd-H5HA, and at 6, 12, 24, 48 and 96 h after inoculation and the animals were euthanized. The lung washes were collected and used to monitor levels of innate cytokines by multiplex assays using a 32-plex Kit from Millipore Sigma. *, significant at p<0.05; **, significant at p<0.01.

**Figure 7:**
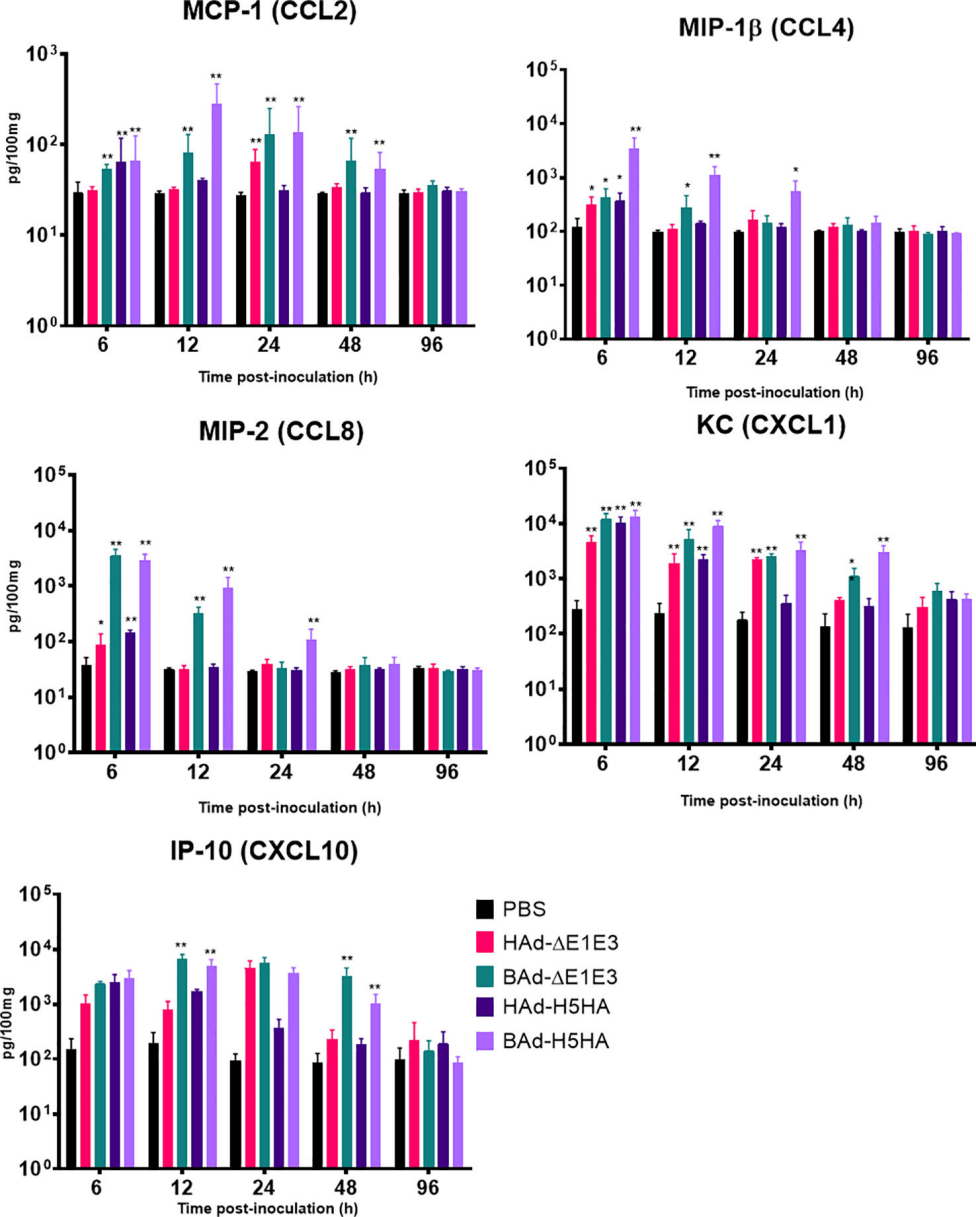
Innate chemokines multiplex assay showing the increase in chemokines levels in BAdV vectors inoculated groups. BALB/c mice (3 animals/group) were inoculated IN once with PBS or with 3 × 10^7^ PFU per animal of HAd-ΔE1E3, BAd-ΔE1E3, HAd-H5HA or BAd-H5HA, and at 6, 12, 24, 48 and 96 h after inoculation and the animals were euthanized. The lung washes were collected to monitor levels of innate chemokines by multiplex assays using a 32-plex Kit from Millipore Sigma. *, significant at p<0.05; **, significant at p<0.01.

To further verify whether there was enhanced induction of innate and adaptive immunity-related factors, cytokines, and chemokines in the lungs of mice inoculated with BAd-H5HA, the lung wash samples from mock-, HAd-ΔE1E3-, BAd-ΔE1E3-, HAd-H5HA-, or BAd-H5HA-inoculated groups were assayed using a 32-multiplex kit assay. There was a transient increase in the levels of IL-1α, IL-1β, IL-5, tumor necrosis factor-alpha (TNF-α), Leukemia inhibitory factor LIF, IL-17, G-CSF, and GM-CSF cytokines in the lung washes of the group inoculated with BAd-H5HA or BAd-ΔE1E3 compared to the group inoculated with HAd-H5HA or HAd-ΔE1E3 (**Figure 6**). There were transient increases in the levels of chemokines CCL2, CCL4, CCL8, CXCL1 and CXCL10 in the lung washes of the group inoculated with BAd-H5HA or BAd-ΔE1E3 compared to the group inoculated with HAd-H5HA or HAd-ΔE1E3 (**Figure 7**). These data suggest that the BAdV vector platform is a better gene delivery system than the HAdV vector in stimulating the innate immune responses in mice.

## Discussion

AdV vectors have enormous potential as a gene delivery platform for developing recombinant vaccines against infectious diseases and cancer immunotherapy (26–32). The concept of preexisting AdV vector immunity in humans (33) has created a niche for developing novel AdV vectors that can overcome the preexisting vector immunity barrier. Along this direction, we developed a BAdV vector platform (34) and demonstrated its utility in eluding an exceptionally high level of vector immunity in a mouse model (35). We have shown that IN immunization of mice with BAd-H5HA elicited significantly higher levels of humoral (including mucosal) and CMI responses at a lower vector dose, resulting in complete protection following challenge with an antigenically distinct influenza virus compared to the HAd-H5HA-immunized group (25). Enhanced immune responses with BAd-H5HA were also observed with the IM route of immunization (25); however, complete protection was conferred with a 30-fold vaccine dose compared to the vaccine dose used for the IN inoculation. These observations led us to pursue the potential factors responsible for high levels of humoral and CMI responses following IN immunization with BAd-H5HA.

Our RNA-Seq analyses of the lung samples from vector-inoculated mice revealed a distinct spectrum and longer duration of DE genes, including genes associated with innate and adaptive immunity in the BAd-H5HA-inoculated group compared to the HAd-H5HA-inoculated group. These results showed that the BAdV vector induced significantly higher levels of several innate and adaptive immunity-related host factors compared to the HAdV vector. Indirectly, these transcriptome analyses also implicate that the BAdV vaccine platform could serve as an excellent gene delivery vehicle for recombinant vaccines. Further studies are needed to investigate the roles of the vital DE genes and their cellular origin in the BAd-H5HA-inoculated group in inducing enhanced immune responses.

Enhanced induction of innate and adaptive immunity-related host factors, cytokines, and chemokines in the lungs of mice inoculated with BAd-H5HA broadly supports the outcomes of transcriptome analyses. It is not a surprise since previously we have demonstrated the enhanced expression of CCL2, CCL3, CCL4, CCL5, CXCL2, TNF-α, CXCL-10, interferon-gamma (IFN-γ), IL-6, TLR2, TLR-3, TLR-4, TLR-7 and TLR9 in the spleen at early time points in BAdV vector-inoculated groups compared to that of HAdV vector-inoculated groups by intravenous (IV) injection (36). It further emphasizes that the BAdV vector platform can induce higher levels of innate and adaptive immunity-related factors, cytokines, and chemokines that could lead to the enhancement of immune responses to BAdV vaccines. This process could significantly impact the development of transgene-specific immune responses.

The BAdV vector stimulated higher expression of TLR2, TLR3, TLR4, TLR7, and TLR9 genes in the lungs than the HAdV vector. These results are aligned with our previously described data (36) where the same TLRs were upregulated in the spleen of the BAdV vector group. TLR-mediated pathways are critical for the activation of transcription factors like interferon regulatory factors (IRFs) and nuclear factor κB (NF-κB), which determine the outcome of the innate immune responses (37). The qRT-PCR analyses of the top ten DE genes identified by RNA-Seq in the lungs of BAd-H5HA confirmed their upregulated state. These genes are involved in natural killer cell pathways, TLR pathways, cytokine-receptor interactions, and viral protein interaction with cytokine, suggesting the advantage of the BAdV vector as a vaccine vector might be explained through its better stimulation of the innate and adaptive immune factors.

In summary, there are enhanced innate and adaptive immunity-related factors in the lungs of mice in the BAdV vector group compared to the HAdV vector group, suggesting the preeminence of the BAdV vaccine platform for developing effective vaccines against emerging infectious diseases and cancer immunotherapy.

## Materials and methods

### Cell lines and Ad vectors

293 (human embryonic kidney cells expressing HAdV5 E1 proteins) (38), BHH3 (bovine-human hybrid clone 3) (39), and BHH2C (bovine-human hybrid clone 2C) (39) cells as monolayer cultures were grown using minimum essential medium (MEM) (Life Technologies, Thermo Fisher Scientific, Waltham, MA) supplemented with either 10% reconstituted fetal bovine serum or fetal calf serum (Hyclone, Thermo Fisher Scientific) and gentamycin (50 µg/ml).

The generation and characterization of BAd-ΔE1E3 (BAdV3 E1 and E3 deleted empty vector) (40), BAd-H5HA [BAd3 E1 and E3 deleted vector expressing HA of A/Hong Kong/156/97(H5N1) (HK/156)] (19), HAd-ΔE1E3 (HAdV5 E1 and E3 deleted empty vector) (41), HAd-H5HA [HAdV5 E1 and E3 deleted vector expressing HA of HK/156] (42), were described earlier. BAd-ΔE1E3 and BAd-H5HA were replicated and titrated in BHH3 cells as described elsewhere (19), whereas HAd-ΔE1E3 and HAd-H5HA were grown in 293 cells and titrated in BHH2C cells as described earlier (43). As described previously, all vectors were purified by cesium chloride density-gradient ultracentrifugation (44, 45).

### Animal inoculation studies

All mouse studies were conducted with the approvals of the Institutional Animal Care and Use Committee (IACUC) and the Institutional Biosafety Committee (IBC). Six-to-eight-week-old BALB/c mice (Envigo RMS, Inc., Indianapolis, IN) were mock-inoculated with phosphate-buffered saline (PBS), pH 7.2 or inoculated IN with 3 × 10^7^ PFU of BAd-ΔE1E3 [4.2 × 10^8^ virus particles (VP)], BAd-H5HA (6.8 × 10^8^ VP), HAd-H5HA (1.2 × 10^9^ VP), or HAd-ΔE1E3 (8.9 × 10^8^ VP). The plaque forming units and virus particle counts were calculated as previously described (46). At 6, 12, 24, 48, and 96 h PI, three animals/group were anesthetized with ketamine-xylazine solution, and the lung washes were prepared by homogenizing one lung from each animal in 1 mL of PBS as described (25, 47). The lung tissue samples were also collected in Invitrogen RNAlater Stabilization Solution (Thermo Fisher Scientific # AM7020) for RNA extraction.

### RNA isolation, sequencing, and analyses

The lung tissue samples in RNAlater were used to extract RNA using Monarch Total RNA Miniprep Kit (Thermo Fisher Scientific #50-152-7886), and the RNA quality was confirmed by 1% agarose gel electrophoresis and Agilent 2100 bioanalyzer 2100 Total RNA Nano Series II. RNA samples were sent for RNA-Seq using 250-300 bp insert cDNA library with Illumina NovaSeq platforms with paired-end 150 bp (PE 150) sequencing strategy using 20 million reads/sample and ≥ 6 G Raw Data/Sample depth (Novogene, Sacramento, CA). The data quality parameters are shown (**Supplementary Table 1**). The data were analyzed by a bioinformatics specialist using a combination of programs, including STAR, HTseq, Cufflink, and wrapped scripts. Alignments were parsed using the Tophat program, and differential expressions were determined through DESeq2/edgeR. GO and KEGG enrichments were implemented by ClusterProfiler. Gene fusion and the difference of alternative splicing events were detected by Star-fusion and rMATS software.

Sequences were quality-checked using FastQC for completeness, depth, and read quality. Sequences were aligned to the mm10 *Mus musculus* reference genome using a STAR aligner (48). Gene quantification was done using HTSeq-count (49).

### Differential gene expression

DESeq2 determines DE genes between at least two experimental groups (50–52). Genes with low counts are filtered for subdued expression by their normalized mean counts, and raw p-values are adjusted for multiple testing using the Benjamini-Hochberg correction. For this experiment, genes considered significantly DE are those with adjusted p-values controlled at False Discovery Rates (FDR) < 0.01 specific threshold. Volcano plots were produced in the Enhanced Volcano R package (53) and showed the un-transformed log (2) fold change of each gene plotted against its adjusted p-value, both of which were calculated in DESEq2. Twenty genes with the highest mean-normalized counts were selected to generate the heatmap.

### Reverse transcription cDNA synthesis

The extracted RNA samples from the lungs were converted to a single complementary DNA strand (cDNA) using High-Capacity cDNA Reverse Transcription Kit (Applied Biosystems, Thermo Fisher Scientific) according to the manufacturing recommendation. 500 nanogram RNA from each sample was converted to cDNA and used in the downstream qPCR.

### Quantitative polymerase reaction

TaqMan Fast Universal PCR 2X Master Mix (Applied Biosystems, Thermo Fisher Scientific) was used to evaluate the differential gene expression between the groups. A real-time PCR was conducted with a 20 µL reaction volume with ROX dye as a passive internal reference to normalize non-PCR-related fluorescence fluctuations. The QuantStudio 3 real-time PCR System (Thermo Fisher, Fisher Scientific) was used to detect differences in each target quantity. TaqMan Gene Expression Assays (Applied Biosystems, Thermo Fisher Scientific) were used to evaluate the differentially expressed TLRs. The 18S ribosomal RNA gene was used as an internal housekeeping gene. The primers and probes of the top ten DE genes identified by RNA-Seq were obtained from TaqMan assays and arrays (Thermo Fisher Scientific) (**Supplementary Table 3**).

### Functional pathways and enrichment analysis

ShinyGo 0.77 (http://bioinformatics.sdstate.edu/go/), the web-based bioinformatics data analysis tool, was used to analyze gene/s functional pathways and process the enrichment analysis for the DE top ten genes at 6, 12, 24, and 48 h PI; *Mus musculus* was used as the input species. The DE genes were annotated and used in the functional enrichment analysis by the default settings for determining the significance of Gene Ontology (GO) Biological Processes and Kyoto Encyclopedia of Genes and Genomes KEGG pathways. The ShinyGO is another web-based data analysis tool used to graphically visualize the upregulated genes’ functions and enrichment results (54). The P-value cutoff set was set at a false discovery rate (FDR) = 0.05.

### Multiplex cytokine analysis

The lung washes were used to monitor levels of innate and adaptive immunity-related factors, cytokines, and chemokines by multiplex assays using Mouse Cytokine/Chemokine Magnetic Bead Panel - Premixed 32 Plex - Immunology Multiplex Assay (Millipore Sigma, St. Louis, MO as described (55). Briefly, the samples were centrifuged at 15,000 g for 10 min, and analyzed for interleukin (IL)-1 alpha, IL-1 beta, IL-2, IL-3, IL-4, IL-5, IL-6, IL-7, IL-9, IL-10, IL-12 (p40), IL-12 (p70), IL-13, IL-15, IL-17, interferon-gamma inducible protein (IP10), keratinocyte-derived chemokine (KC), eotaxin, monocyte chemoattractant protein 1 (MCP-1), monokine-induced by gamma interferon (MIG), macrophage inflammatory protein-2 alpha (MIP-2α), macrophage inflammatory protein-1 alpha (MIP-1α), macrophage inflammatory protein-1 beta (MIP-1β), IFN-γ, lipopolysaccharide-induced CXC chemokine (LIX), TNF-α, regulated upon activation normal T cell expressed and secreted (RANTES), leukemia inhibitory factor (LIF), granulocyte colony-stimulating factor (G-CSF), granulocyte-macrophage colony-stimulating factor (GM-CSF), macrophage colony-stimulating factor (M-CSF), vascular endothelial growth factor (VEGF). Samples were 2-fold diluted and incubated with the pre-mixed capture antibody-coupled beads in 96-well plates at 4°C overnight. The beads were washed and incubated with the biotinylated secondary antibodies for 2 h. Streptavidin–phycoerythrin was added and incubated for 30 min, and the beads were washed and resuspended in sheath fluid. The standard curve range was 3.2-10,000 pg/ml. The analysis was performed by a Bio-Plex 200 System with High Throughput Fluidics (HTF) Multiplex Array System (Indiana University Cancer Center Facility, Indianapolis, IN).

### Statistical analyses

One and two-way ANOVA with Bonferroni post-test were performed to determine statistical significance for the multiplex assay. The *p-*value below 0.05 was considered statistically significant.

## Data availability statement

The datasets presented in this study are deposited in NCBI BioProject accession number: PRJNA1039157.

## Ethics statement

The animal study was approved by Institutional Animal Care and Use Committee (IACUC) and the Institutional Biosafety Committee (IBC). The study was conducted in accordance with the local legislation and institutional requirements.

## Author contributions

ES: Formal Analysis, Investigation, Methodology, Validation, Visualization, Writing – original draft, Writing – review & editing. NE: Formal Analysis, Investigation, Methodology, Software, Writing – review & editing. GZ: Conceptualization, Supervision, Validation, Writing – review & editing. SM: Investigation, Supervision, Writing – review & editing. SS: Conceptualization, Formal Analysis, Supervision, Writing – review & editing. SM: Conceptualization, Formal Analysis, Funding acquisition, Supervision, Validation, Writing – review & editing.
