# Supplementary material for: Enhancement of mucosal innate and adaptive immunity following intranasal immunization of mice with a bovine adenoviral vector
Source: Front Immunol. 2023 Nov 21;14:1305937. doi: 10.3389/fimmu.2023.1305937 (PMC10702558; doi:10.3389/fimmu.2023.1305937)
Supplement: Supplementary file 1 [file Presentation_1.pdf]

Supplementary Data

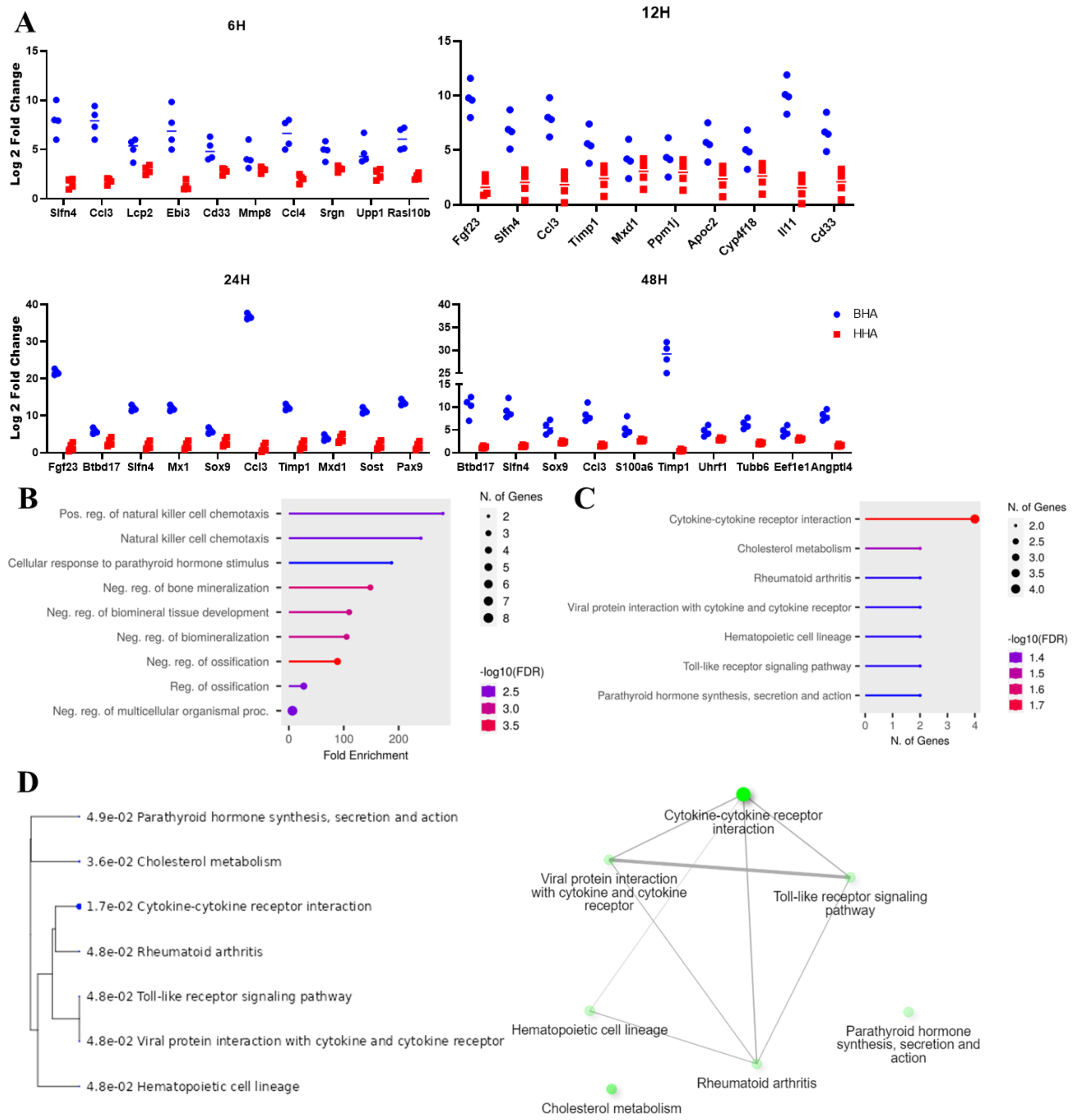

Fig. S1. Quantitative reverse transcriptase polymerase chain reaction (qRT-PCR) to validate upregulation of top ten differentially expressed (DE) genes (Table S2) and their pathways. **(A)** RNA samples from two mice/group infected with BAd-H5HA (BHA) or HAd-H5HA (HHA) at 6, 12, 24, and 48 h post-inoculation were used for qRT-PCR. Log2 fold changes in expression levels of top ten RNASeq DE genes between BHA and HHA groups. **(B)** A lollipop blot chart of the gene ontology enrichment analysis of the biological pathways involved in the upregulated gene list of the BHA group using ShinyGO 0.77. **(C)** A lollipop blot chart of the gene ontology enrichment analysis of the KEGG pathways involved in the

upregulated gene list of the BHA group using ShinyGO 0.77. **(D)** A tree map (right) and network map (left) show the KEGG pathway interactions.

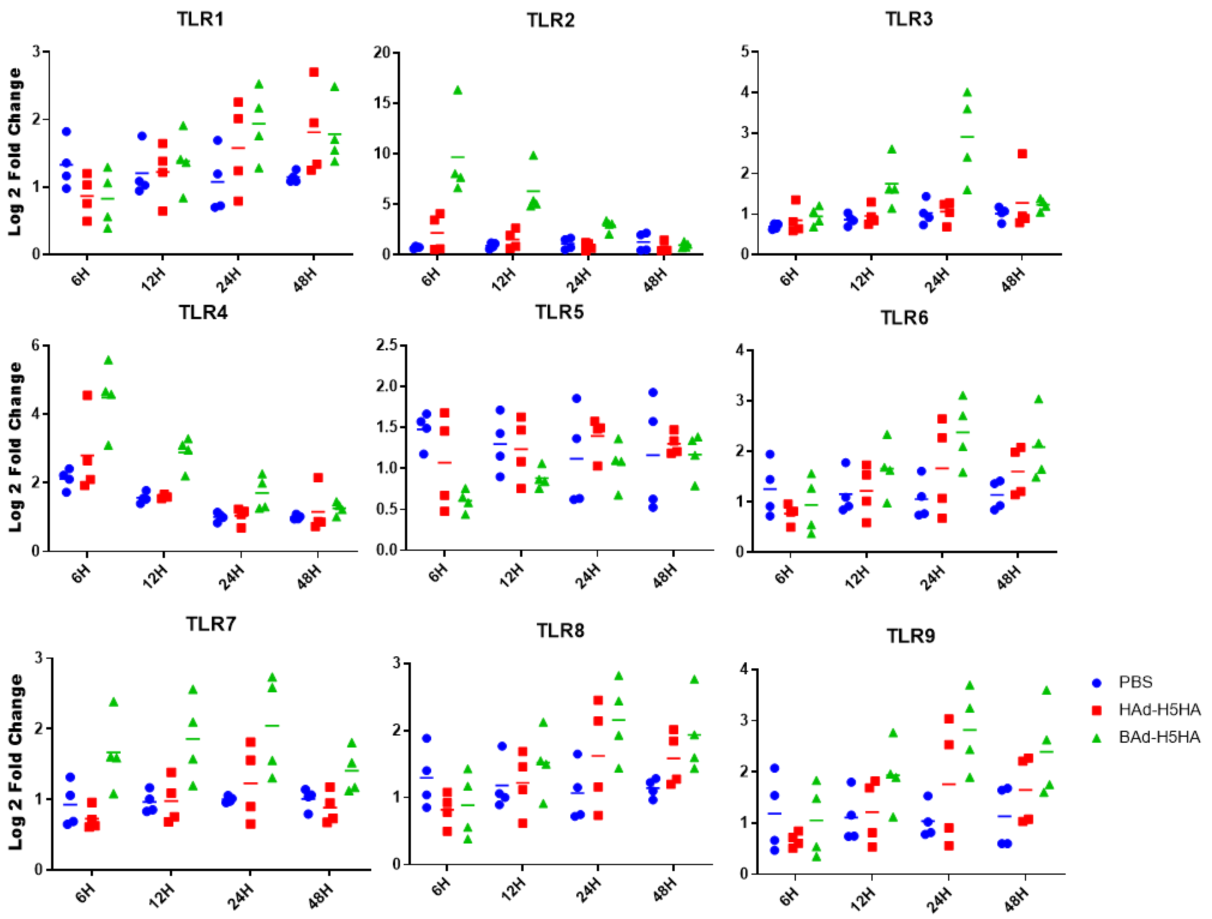

Fig. S2. Quantitative reverse transcriptase polymerase chain reaction (qRT-PCR) of Toll-like receptors gene expression levels in the lungs of BAd-H5HA, HAd-H5HA, or mock (PBS) inoculated mice at 6, 12, 24, and 48 h post-inoculation (PI). The expression differences in log2 fold changes are shown. The 18S ribosomal RNA gene expression was used to normalize the data.

Table S1. Summary of RNA-Seq data quality.

Sample name	Raw reads	Clean reads	Raw bases	Clean bases	Error rate(%)	Q20(%)	Q30(%)	GC content(%)
BHA_1_06	42918428	40929318	6.4G	6.1G	0.03	97.42	93.26	49.64
BHA_1_12	43368598	41888446	6.5G	6.3G	0.03	97.17	92.68	49.87
BHA_1_24	43941642	41981168	6.6G	6.3G	0.03	97.37	93.17	50.54
BHA_1_48	43394882	41421514	6.5G	6.2G	0.03	97.27	92.96	50.21
BHA_2_06	47103238	44479998	7.1G	6.7G	0.03	97.29	93.04	49.67
BHA_2_12	45539990	43629470	6.8G	6.5G	0.03	97.14	92.68	49.83
BHA_2_24	49721160	46754026	7.5G	7G	0.03	97.45	93.36	50.33
BHA_2_48	45470372	43336754	6.8G	6.5G	0.03	97.4	93.22	50.08
HHA_1_06	50530026	47369324	7.6G	7.1G	0.03	97.3	93.03	49.75
HHA_1_12	45207908	42576504	6.8G	6.4G	0.03	97.24	92.89	50.15
HHA_1_24	50264232	48566598	7.5G	7.3G	0.03	97.24	92.92	49.91
HHA_1_48	47682200	44950874	7.2G	6.7G	0.03	97.37	93.17	49.78
HHA_2_06	49711260	47483208	7.5G	7.1G	0.03	97.34	93.17	49.91
HHA_2_12	61716310	57245500	9.3G	8.6G	0.03	97.55	93.57	50.11
HHA_2_24	55797986	53157256	8.4G	8G	0.03	97.45	93.36	49.94
HHA_2_48	47981058	46011258	7.2G	6.9G	0.03	97.35	93.12	49.65
PBS_1_06	44439868	41794268	6.7G	6.3G	0.03	97.35	93.14	49.87
PBS_1_12	45362076	42890622	6.8G	6.4G	0.03	97.52	93.44	49.99
PBS_1_24	53209464	51309256	8G	7.7G	0.03	97.45	93.32	49.9
PBS_1_48	46208582	43527072	6.9G	6.5G	0.03	97.11	92.64	49.85
PBS_2_06	41834946	39442854	6.3G	5.9G	0.03	97.53	93.52	49.95
PBS_2_12	45995038	44356058	6.9G	6.7G	0.03	97.33	93.12	49.62
PBS_2_24	50534750	48158686	7.6G	7.2G	0.03	97.09	92.57	49.4
PBS_2_48	49474892	47115064	7.4G	7.1G	0.03	97.38	93.2	49.78

Table S2. List of top DE genes in the BAd-H5HA-inoculated group compared to the HAd-H5HA group.

A. **List of top DE genes in the BAd-H5HA-inoculated group compared to the HAd-H5HA group at 6 hr.**

Gene ID	BHA (Read Count)	HHA (Read Count)	Log 2 Fold Change	P value	P adj	Gene Name	Gene Description
ENSMUSG00000000204	455.92	57.52	2.99	1.62E-11	8.17E-08	Slfn4	schlafen_4
ENSMUSG00000000982	50.79	6.94	2.87	3.38E-10	6.28E-07	Ccl3	chemokine_(C-C_motif)_ligand_3
ENSMUSG00000002699	100.90	27.55	1.87	1.32E-05	4.05E-03	Lcp2	lymphocyte_cytosolic_protein_2
ENSMUSG00000003206	5.11	0.66	2.94	5.38E-07	3.22E-04	Ebi3	Epstein-Barr_virus_induced_gene_3
ENSMUSG00000004609	215.59	51.24	2.07	1.40E-06	6.90E-04	Cd33	CD33_antigen
ENSMUSG00000005800	101.84	25.95	1.97	4.90E-06	1.92E-03	Mmp8	matrix_metallopeptidase_8
ENSMUSG00000018930	31.14	5.56	2.48	5.89E-08	5.59E-05	Ccl4	chemokine_(C-C_motif)_ligand_4
ENSMUSG00000020077	552.52	147.38	1.91	7.29E-06	2.62E-03	Srgn	serglycin
ENSMUSG00000020407	76.08	16.53	2.20	4.91E-07	3.09E-04	Upp1	uridine_phosphorylase_1
ENSMUSG00000020684	53.61	10.47	2.36	1.14E-07	8.71E-05	Rasl10b	RAS-like_family_10_member_B

B. List of top DE genes in the BAd-H5HA-inoculated group compared to the HAd-H5HA group at 12 hr.

Gene ID	BHA (Read Count)	HHA (Read Count)	Log 2 Fold Change	P value	P adj	Gene Name	Gene Description
ENSMUSG00000000182	6.92	0.72	3.25	1.81E-08	8.88E-06	Fgf23	fibroblast_growth_factor_23
ENSMUSG00000000204	1661.68	248.02	2.74	3.70E-10	3.84E-07	Slfn4	schlafen_4
ENSMUSG00000000982	169.88	21.75	2.97	3.39E-11	4.99E-08	Ccl3	chemokine_(C-C_motif)_ligand_3
ENSMUSG00000001131	393.64	72.95	2.43	2.06E-08	9.67E-06	Timp1	tissue_inhibitor_of_metalloproteinase_1
ENSMUSG00000001156	591.08	147.90	2.00	2.76E-06	5.73E-04	Mxd1	MAX_dimerization_protein_1
ENSMUSG00000002228	16.92	4.10	2.04	1.16E-05	1.80E-03	Ppm1j	protein_phosphatase_1J
ENSMUSG00000002992	4.31	0.78	2.45	3.10E-05	3.92E-03	Apoc2	apolipoprotein_C-II
ENSMUSG00000003484	53.69	11.08	2.28	2.80E-07	8.65E-05	Cyp4f18	cytochrome_P450_family_4_subfamily_f_polypeptide_18
ENSMUSG00000004371	4.77	0.48	3.29	1.26E-07	4.48E-05	Il11	interleukin_11
ENSMUSG00000004609	256.61	39.70	2.69	9.67E-10	8.12E-07	Cd33	CD33_antigen

C. **List of top DE genes in the BAd-H5HA-inoculated group compared to the HAd-H5HA group at 24 hr.**

Gene ID	BHA (Read Count)	HHA (Read Count)	Log 2 Fold Change	P value	P adj	Gene Name	Gene Description
ENSMUSG00000000182	2.14	0.00	8.22	4.11E-08	1.07E-05	Fgf23	fibroblast_growth_factor_23
ENSMUSG00000000202	5.55	1.01	2.46	8.34E-06	9.52E-04	Btbd17	BTB_(POZ)_domain_containing_17
ENSMUSG00000000204	470.11	40.27	3.54	7.27E-15	1.17E-11	Slfn4	schlafen_4
ENSMUSG00000000386	170.74	14.61	3.55	1.22E-14	1.73E-11	Mx1	MX_dynamin-like_GTPase_1
ENSMUSG00000000567	3.53	0.64	2.46	5.88E-05	4.49E-03	Sox9	SRY_(sex_determining_region_Y)-box_9
ENSMUSG00000000982	69.52	1.91	5.18	2.61E-23	2.64E-19	Ccl3	chemokine_(C-C_motif)_ligand_3
ENSMUSG00000001131	177.80	14.92	3.57	8.33E-15	1.28E-11	Timp1	tissue_inhibitor_of_metalloproteinase_1
ENSMUSG00000001156	213.34	57.79	1.88	9.80E-06	1.10E-03	Mxd1	MAX_dimerization_protein_1
ENSMUSG00000001494	4.66	0.42	3.44	5.94E-08	1.46E-05	Sost	sclerostin
ENSMUSG00000001497	1.32	0.00	7.53	8.28E-06	9.48E-04	Pax9	paired_box_9

D. **List of top DE genes in the BAd-H5HA-inoculated group compared to the HAd-H5HA group at 48 hr.**

Gene ID	BHA (Read Count)	HHA (Read Count)	Log-2 Fold Change	P value	P adj	Gene Name	Gene Description
ENSMUSG00000000202	4.45	0.46	3.26	2.23E-07	3.67E-05	Btbd17	BTB_(POZ)_domain_containing_17
ENSMUSG00000000204	390.50	49.87	2.97	2.35E-11	1.13E-08	Slfn4	schlafen_4
ENSMUSG00000000567	9.21	1.95	2.24	8.33E-06	7.87E-04	Sox9	SRY_(sex_determining_region_Y)-box_9
ENSMUSG00000000982	28.07	4.01	2.81	2.24E-09	6.86E-07	Ccl3	chemokine_(C-C_motif)_ligand_3
ENSMUSG00000001025	627.67	158.90	1.98	3.29E-06	3.67E-04	S100a6	S100_calcium_binding_protein_A6_(calcyclin)
ENSMUSG00000001131	255.91	8.42	4.92	1.52E-23	4.86E-20	Timp1	tissue_inhibitor_of_metalloproteinase_1
ENSMUSG00000001228	102.07	28.46	1.84	1.80E-05	1.49E-03	Uhrf1	ubiquitin-like_containing_PHD_and_RING_finger_domains_1
ENSMUSG00000001473	181.28	34.87	2.38	4.78E-08	9.86E-06	Tubb6	tubulin_beta_6_class_V
ENSMUSG00000001707	27.82	7.79	1.84	3.78E-05	2.61E-03	Eef1e1	eukaryotic_translation_elongation_factor_1_epsilon_1
ENSMUSG00000002289	212.27	30.06	2.82	1.99E-10	8.07E-08	Angptl4	angiopoietin-like_4

Table S3. List of top DE genes in the BAd-H5HA-inoculated group compared to HAd-H5HA group Gene ID and Part No.

Gene ID	Part No	Gene ID	Part No; Reference
Slfn4	4331182 Mm01298330_m1	Mx1	4331182; Mm00487796_m1
Ccl3	4331182 Mm00441259_g1	Sox9	4331182; Mm00448840_m1
Lcp2	4331182 Mm01187570_m1	Sost	4331182; Mm00470479_m1
Ebi3	4331182 Mm00469294_m1	Pax9	4331182; Mm00440629_m1
Cd33	4331182 Mm00491152_m1	S100a6	4331182; Mm00771682_g1
Mmp8	4331182 Mm00439509_m1	Uhrf1	4331182; Mm00477872_m1
Ccl4	4331182 Mm00443111_m1	Tubb6	4331182; Mm00660543_m1
Srgn	4331182 Mm01169070_m1	Eef1e1	4331182; Mm01349382_m1
Upp1	4331182 Mm00447676_m1	Angptl4	4331182; Mm00480431_m1
Rasl10b	4331182 Mm01302429_m1	Ppm1j	4331182; Mm00712588_m1
Fgf23	4331182 Mm01183126_m1	Apoc2	4331182; Mm00437571_m1
Timp1	4331182 Mm01341361_m1	Cyp4f18	4351372; Mm07298284_m1
Mxd1	4331182 Mm00487504_m1	Il11	4331182; Mm00434162_m1
Btbd17	4331182 Mm01248712_m1	18S	4331182; Mm03928990_g1
